# Consensus and variations in cell line specificity among human metapneumovirus strains

**DOI:** 10.1371/journal.pone.0215822

**Published:** 2019-04-23

**Authors:** Naganori Nao, Ko Sato, Junya Yamagishi, Maino Tahara, Yuichiro Nakatsu, Fumio Seki, Hiroshi Katoh, Aiko Ohnuma, Yuta Shirogane, Masahiro Hayashi, Tamio Suzuki, Hideaki Kikuta, Hidekazu Nishimura, Makoto Takeda

**Affiliations:** 1 Department of Virology 3, National Institute of Infectious Diseases, Musashimurayama, Tokyo, Japan; 2 Virus Research Center, Clinical Research Division, Sendai Medical Center, Sendai, Japan; 3 Reserach Center for Zoonosis Control, Hokkaido University, Sapporo, Japan; 4 Department of Dermatology, Faculty of Medicine, Yamagata University, Yamagata, Japan; 5 Pediatric Clinic, Touei Hospital, Sapporo, Japan; Kliniken der Stadt Köln gGmbH, GERMANY

## Abstract

Human metapneumovirus (HMPV) has been a notable etiological agent of acute respiratory infection in humans, but it was not discovered until 2001, because HMPV replicates only in a limited number of cell lines and the cytopathic effect (CPE) is often mild. To promote the study of HMPV, several groups have generated green fluorescent protein (GFP)-expressing recombinant HMPV strains (HMPV^GFP^). However, the growing evidence has complicated the understanding of cell line specificity of HMPV, because it seems to vary notably among HMPV strains. In addition, unique A2b clade HMPV strains with a 180-nucleotide duplication in the G gene (HMPV A2b_180nt-dup_ strains) have recently been detected. In this study, we re-evaluated and compared the cell line specificity of clinical isolates of HMPV strains, including the novel HMPV A2b_180nt-dup_ strains, and six recombinant HMPV^GFP^ strains, including the newly generated recombinant HMPV A2b_180nt-dup_ strain, MG0256-EGFP. Our data demonstrate that VeroE6 and LLC-MK2 cells generally showed the highest infectivity with any clinical isolates and recombinant HMPV^GFP^ strains. Other human-derived cell lines (BEAS-2B, A549, HEK293, MNT-1, and HeLa cells) showed certain levels of infectivity with HMPV, but these were significantly lower than those of VeroE6 and LLC-MK2 cells. Also, the infectivity in these suboptimal cell lines varied greatly among HMPV strains. The variations were not directly related to HMPV genotypes, cell lines used for isolation and propagation, specific genome mutations, or nucleotide duplications in the G gene. Thus, these variations in suboptimal cell lines are likely intrinsic to particular HMPV strains.

## Introduction

Human metapneumovirus (HMPV) is a major causative agent of acute respiratory infections especially in young children, older people, and patients with underlying conditions such as cardiopulmonary diseases and diabetes [1–3]. The virus is a member of the family *Pneumoviridae* and has a non-segmented negative sense RNA genome containing 8 genes in the order: 3′-N-P-M-F-M2-SH-G-L-5′. The genome encodes 9 viral proteins including three surface glycoproteins: F (fusion), SH (small hydrophobic), and G (glycol-) proteins. HMPV has been circulating worldwide for more than 6 decades [4], and about half of children are infected with HMPV before 2 years of age, and most children are infected before 5 years of age [4]. HMPV is classified into two antigenically distinct groups, A and B. Based on nucleotide sequence variations, each group is further divided into two subgroups (A1 and A2 in group A, and B1 and B2 in group B) [5, 6]. Furthermore, in the A2 subgroup there are two phylogenetically distinct clades, A2a and A2b [7]. In addition, currently unique A2b clade HMPV strains with a 180- or 111-nucleotide duplication (180nt-dup and 111nt-dup, respectively) in the G gene have been detected [8–10]. Although antigenic variations may contribute to repeat HMPV infections, antigenic changes are not required for HMPV to cause symptomatic reinfection, because HMPV infection usually does not result in lifelong protective immunity [4, 11].

Many characteristics of viruses have been identified with isolated viruses in cultured cells. However, phenotypes of isolated viruses may differ from those of the original viruses circulating in patients, because viruses may be selected or acquire mutations during isolation and propagation in specific cell lines [12–15]. Despite its great impact on human health and the wide spread of the disease, HMPV was not discovered until 2001 [4] partly owing to the difficulty of HMPV detection in cultured cells [16–19]. HMPV replicates only in a limited number of cell lines and was initially isolated using tertiary monkey kidney (tMK) cells [4]. Also, the virus requires trypsin to be cultivated in cell lines [4]. The cytopathic effect (CPE) is often mild and needs to be present for at least 2 weeks to be detected, even when the culture media are supplemented with trypsin [1, 16–19]. Recently, the human malignant melanoma MNT-1 cell line has been demonstrated to have a clear CPE upon infection with HMPV [16]. To promote the study of HMPV, several groups have generated green fluorescent protein (GFP)-expressing recombinant HMPV (HMPV^GFP^) strains [20–23]. Previously our group has also generated a HMPV^GFP^ strain based on the clinical isolate JPS02-76 strain, which was isolated using tMK cells [11, 21]. As expected, the recombinant JPS02-76 strain (JPS02-76^GFP^) has shown high infectivity only in a few cell lines, such as LLC-MK2 and Vero cells [21]. These observations are consistent with previous findings showing that LLC-MK2 and Vero cells are the most useful cell lines for the isolation of HMPV [1, 3, 18, 19, 24–28]. Abiko et al. (2007) [19] showed that VeroE6 cells were better than LLC-MK2 cells for isolation of HMPV. In addition to LLC-MK2 and Vero cells, human bronchial epithelial BEAS-2B cells have recently been used for HMPV studies [29–32]. In certain studies, lung adenocarcinoma A549 cell line [30, 32] and human bronchial epithelial 16HBE cell line [32] were also used. Landry et al. [33] reported similar infectivity of LLC-MK2, A549 and HEp-2 cell lines with HMVP, but A549 cells are very poorly infected with our JPS02-76^GFP^ recombinant strain [21]. The original study [4] describing the discovery of HMPV also demonstrated poor infectivity of A549 cells with HMPV. High infectivity of HEp-2 cells was reported by Chan et al. [34], while low infectivity of HEp-2 cells was documented in other studies [1, 27]. Therefore, the growing evidence has resulted in a rather complicated situation for understanding the cell line specificity of HMPV. Therefore, in this study we re-evaluated and compared the cell line specificity of recently isolated clinical strains of HMPV, including novel A2b subtype strains with 180nt-dup (A2b_180nt-dup_), five recombinant HMPV^GFP^, which have been used in different laboratories, and the newly generated recombinant HMPV A2b_180nt-dup_ strain, MG0256-EGFP, to advance our understanding of HMPV and reinforce the value of previous HMPV study data.

## Materials and methods

### Cell lines

Vero (ATCC CCL-81), VeroE6 (ATCC CRL-1586), and HeLa229 (ATCC CCL-2.1) cells were grown in Dulbecco’s modified Eagle’s medium (DMEM) supplemented with 5% fetal calf serum (FCS) and antibiotics (100 U/ml penicillin and 0.1 mg/ml streptomycin). MNT-1 [35] and HEK293 (ATCC CRL-1573) cells were grown in DMEM supplemented with 10% FCS and antibiotics. LLC-MK2 derivative cells (ATCC CCL-7.1) were grown in Eagle’s minimum essential medium supplemented with 10% FCS and antibiotics. A549 (NIHS JCRB0076) and BEAS-2B (ATCC CRL-9609) cells were grown in a 1:1 mixture of DMEM and Ham’s F-12 medium supplemented with 10% FCS and antibiotics. All cells were incubated at 37°C in a 5% CO_2_ atmosphere except where otherwise indicated. Generally, trypsin is used for HMPV propagation in cultured cells. Trypsin activates proteolytically the HPMV F protein. Our group has previously demonstrated that transmembrane serine protease 2 (TMPRSS2) also activates the HMPV F protein [21]. To construct TMPRSS2-expressing VeroE6 cells, the coding sequence of human TMPRSS2 [36] was inserted into an expression vector pAP3neo (Takara Bio). VeroE6 cells were transfected with pAP3neo encoding human TMPRSS2 and cultured in the presence of 1.0 mg/ml geneticin (G418; Nacalai Tesque). A transduced VeroE6 cell line was cloned by limiting dilution to enrich the population of TMPRSS2-expressing VeroE6 cells (VeroE6/TMPRSS2). The cloned TMPRSS2-expressing VeroE6 (VeroE6/TMPRSS2) cells were grown in DMEM supplemented with 5% FCS and antibiotics. To obtain influenza virus NS1-expressing A549 cells, the coding sequence of influenza virus NS1 protein (PR8 strain) was FLAG-tagged and inserted into a murine leukemia virus-based retroviral vector, pMXs-TRES-Puro Retroviral Vector (pMXs-IP) (Cell Biolabs). To generate the retrovirus vector, Platinium-GP cells (Cell Biolabs) were cotransfected with pMXs-IP encoding FLAG-tagged influenza virus NS1 protein and an expression plasmid for vesicular stomatitis virus G protein by using TransIT-LT1 (Mirus Bio), according to the manufacturer’s instructions. Three days later, retrovirus vectors in the culture supernatants were collected, and A549/hSLAM cells [37] were infected with the retrovirus vectors. A549/hSLAM cells stably expressing influenza virus NS1 protein (A549/hSLAM-NS1) were selected in medium containing 5 μg/ml puromycin (Sigma-Aldrich).

### Quantitative RT-PCR assay

Total RNA was extracted from vesicular stomatitis virus, New Jersey strain, (VSV)-infected or mock-infected A549/hSLAM and A549/hSLAM-NS1 cells using TRIzol LS (Life Technologies), and first-strand cDNA was synthesized using SuperScript IV reverse transcriptase (Thermo Fisher Scientific) with Oligo(dt)20 Primer (5’-TTTTTTTTTTTTTTTTTTTT-3’), according to the manufacturer’s instructions. The amount of each mRNA was measured using the Universal Probe Library and the Light Cycler 480 system (Roche) and normalized to that of hypoxanthine phosphoribosyltransferase 1 (HPRT1) mRNA, in a similar manner as previously described [38]. Primers and Universal ProbeLibrary (UPL) probes (Roche) used for quantitative PCR (qPCR) are shown in supporting information (S1 Table).

### Clinical samples and clinical isolates of HMPV strains

Clinical samples (throat swabs and nasal secretions) collected from patients with symptoms of acute respiratory infections in Sendai city, Japan [16] were used. Isolation procedures and clinical isolates of HMPV strains have also been previously reported [16]. The subtypes of HMPV strains were determined as previously described [39]. Two HMPV A1 strains, IA3-2002 and IA10-2003, were obtained from ZeptoMetrix (https://www.zeptometrix.com/) and propagated in VeroE6/TMPRSS2 cells.

### Construction of the full-length HMPV A2b_180nt-dup_ genome cDNA plasmid encoding EGFP

The full-length genome cDNA of the hMPV/Sendai/0256/2015 strain flanked by the T7 promoter and hepatitis delta virus ribozyme (Rz) sequence was cloned into pBluescript vector (Stratagene). An additional transcription unit for EGFP was created between the N and P genes. Cloning was performed by using In-Fusion HD Cloning Kit (Clontech) according to the manufacturer’s instructions. The constructed plasmid was sequenced to confirm the absence of unexpected mutations. The recombinant hMPV/Sendai/0256/2015 strain expressing EGFP was designated as MG0256-EGFP based on the prefecture code of isolated location (MG; Miyagi prefecture) and the ID code for the original isolate.

### GFP-expressing recombinant HMPV strains

The GFP-expressing recombinant CAN97-83 strain (rHMPV-GFP) [20] was obtained from Vira Tree (http://www.viratree.com/) and propagated in VeroE6/TMPRSS2 cells. GFP-expressing recombinant JPS02-76 [21], JPN03 (rHMPV-Rluc/GFP) [22], NL/1/99 (NL/1/99-gfp) [23], NL/1/00 (NL/1/00-gfp) [23], and MG0256-EGFP (this study) strains were generated as previously described [21] and propagated in VeroE6/TMPRSS2 cells. The reagent for rHMPV-Rluc/GFP was kindly provided by Dr. Bin Gotoh (Shiga University of Medical Science). The reagents for NL/1/99-gfp and NL/1/00-gfp were kindly provided by Dr. Ron A.M. Fouchier (Erasmus MC, The Netherlands).

### Viral genome sequences

Total RNAs in clinical samples were extracted using QIamp Viral RNA mini kit (Qiagen), according to the manufacturer’s protocol. Viral RNAs in the extracted RNAs were reverse-transcribed into cDNAs using SuperScript IV Reverse Transcriptase (Thermo Fisher Scientifics) and the viral gene specific primer HMPV_F_gene_1_F_long (5’-GGGACAARTRAAAATGTCTTGGAAAGTGRTG-3’), according to the manufacturer’s protocol. A region spanning the F, M2, SH, and G genes of the viral genome (F-M2-SH-G region) was amplified by PCR with KOD FX Neo (TOYOBO) and a pair of viral gene specific primers, HMPV_F_gene_1_F_long and HMPV_7455R (5’-CCAATCACATATCATAYTTAAYTTKAGAGWGC-3’), according to the manufacturer’s protocol. The amplified DNAs of the F-M2-SH-G region were fragmented and tagged with index adaptors by using NEBNext dsDNA Fragmentase and NEBNext Ultra II DNA Library Prep Kit for Illumina (New England BioLabs), according to the manufacturer’s protocol. Prepared libraries were verified with gel electrophoresis and quantified with Quantus Fluorometer (Promega) before loading on the sequencing chip. Then the index libraries were pooled and sequenced using a MiSeq v3 600-cycle kit (Illumina) to perform 300-bp paired-end sequencing on a MiSeq instrument (Illumina), according to the manufacturer’s protocol. After the sequencing run, reads with the same index sequences were grouped together and subjected to de novo assembly using CLC Genomics Workbench (CLC Bio). Reads were analyzed with the default setting, except for trimming (Quality score: >20; read length: >200 base).

For a full genome analysis of HMPV strains, the total RNA was extracted from HMPV-infected cells using TRIzol LS Reagent (Thermo Fisher Scientifics). Viral RNAs were reverse-transcribed into cDNAs using SuperScript IV Reverse Transcriptase (Thermo Fisher Scientifics) and the viral gene specific primer HMPV_full2_A_F1 (5’-CGACGCGAAAAAAACGCGTATAAATTAARTTAC-3’), according to the manufacturer’s protocol. The first and latter halves of synthesized cDNA were amplified separately using PCR with PrimeSTAR GXL DBA Polymerase (TaKaRa Bio) and two pairs of viral gene specific primers, HMPV_full2_A_F1 and GNR2_mod2 (5’-ACTCCTTTAAGRTACGAATCAGGGAGATAGAC-3’) and HMPV_A2b_G_ORF_1F (5’-ATGGAGGTGAARGTAGRGAACATTCGAGC-3’) and HMPV_full2_A_R13335 (5’-ACGGCAAAAAAACCGTATACATTCAATTATAATTTC-3’). Sequencing libraries were prepared from the amplified DNA by using SMRTBell template prep kit 1.0 reagents (Pacific Biosciences) without fragmentation. The libraries were sequenced on a PacBio RSII instrument with the DNA sequencing reagent 4.0 (Pacific Biosciences) and SMRT Cell 8 Pac V3 (Pacific Biosciences). After the sequencing run, the consensus sequence of each HMPV strain was de novo assembled using Canu software [40]. Sequence data analyzed in this study is shown in supporting information (S1 and S2 Datasets).

### Phylogenetic analysis

Multiple-sequence alignments were constructed with the MAFFT software (ver. 7.123b) using the default settings [41]. Phylogenetic analysis was performed with the maximum likelihood methods in the MEGA software (ver. 7.0.21) [42]. The statistical significance of the tree topologies was tested with bootstrapping (100 replicates).

### Titration of HMPV strain using various cell lines (infectivity assay)

The infectivity titer of viruses may differ greatly when different cell lines are used for titration. First, the infectivity titer (infectious unit [IU]) of working stocks of clinical isolates and GFP-expressing recombinant HMPV strains was determined using VeroE6 cells. For this, VeroE6 cells were incubated with serially diluted virus stocks in DMEM supplemented with 5% FCS and antibiotics at 33°C in a 5% CO_2_ atmosphere. No trypsin was added to the culture media to prevent the second round of infection. At 48 hours post-infection (hpi), the number of cells infected with GFP-expressing recombinant HMPV strain was counted under fluorescent microscopy. At 72 hpi, cells infected with clinical isolates of HMPV strains were subjected to an indirect immunofluorescent assay with mouse anti-HMPV-F monoclonal antibody (clone 1G3) [43] and Alexa Fluor 488-conjugated goat anti-Mouse IgG antibody (Thermo Fisher Science), and the number of HMPV-infected cells was counted under fluorescent microscopy. Based on these data, IU was calculated. Next, seven cell lines (LLC-MK2, Vero-ATCC, HeLa, MNT-1, HEK293, BEAS-2B, and A549) were infected with 100 IU of HMPV strains and incubated in appropriate culture medium without trypsin at 33°C in a 5% CO_2_ atmosphere. At 48 hpi (recombinant HMPV^GFP^ strains) or 72 hpi (clinical isolates), the numbers of HMPV-infected cells were counted as described above to assess the infectivity in these cell lines.

### Ethical considerations

The research proposal was approved by the ethics committee of the Sendai Medical Center (#26–03) and the ethics committee of the National Institute of Infectious Diseases (#873). The consents of participants were obtained verbally. When participants were under 20 years old, consents were obtained from parents or guardians.

## Results

### Isolation of HMPV A2b_180nt-dup_ strains and the phylogenetic relationship among HMPV strains

**Fig 1:**
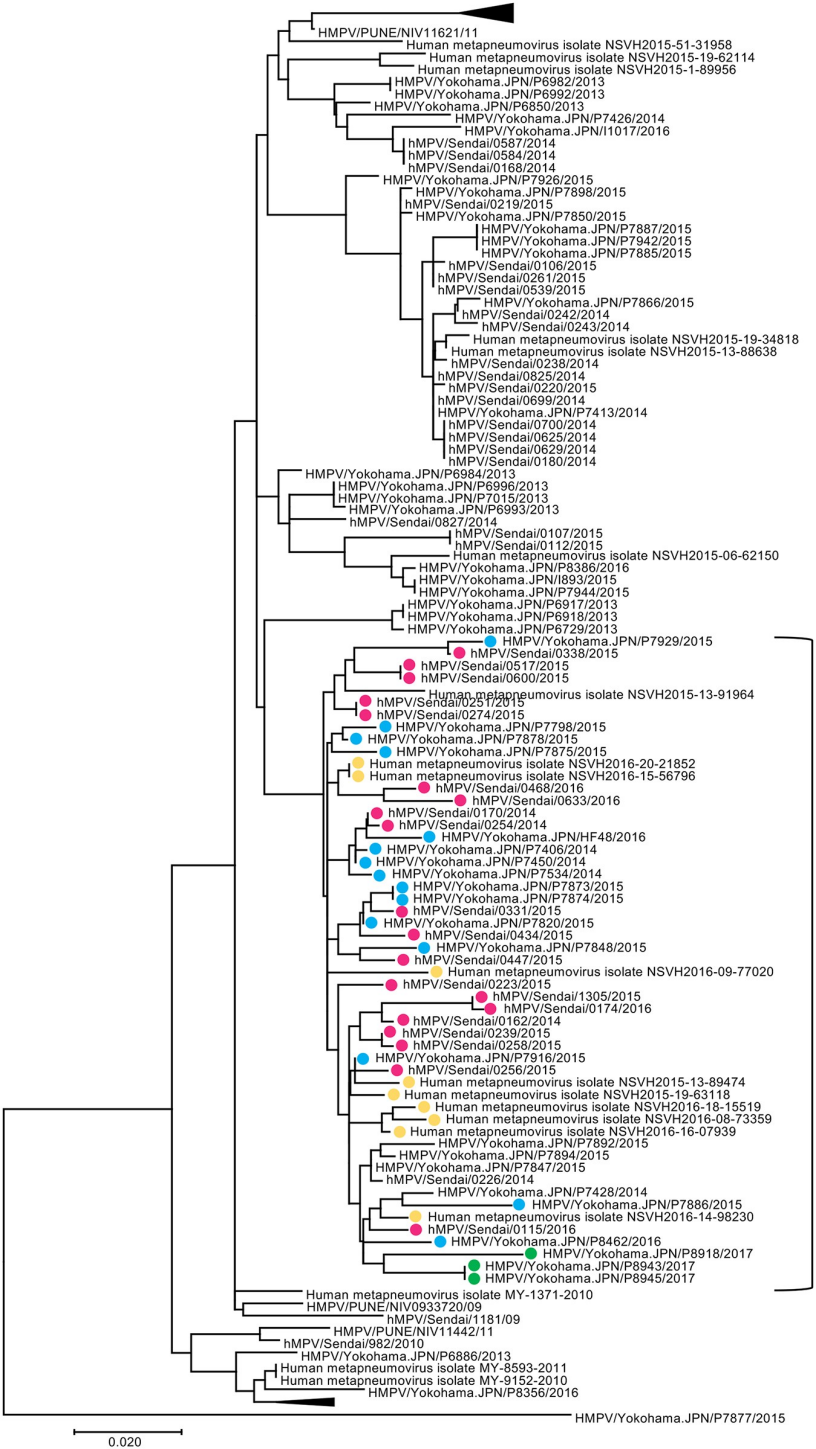
Phylogenetic tree of HMPV G gene sequence. A phylogenetic tree was constructed using the G gene sequences of 41 A2b HMPV strains detected in Sendai and 97 strains obtained from the NCBI nucleotide sequence database. The tree was rooted with A2a HMPV strain HMPV/Yokohama.JPN/P7877/2015 and tested with bootstrapping (100 replicates). HMPV A2b_180nt-dup_ strains detected in Sendai, HMPV A2b_180nt-dup_ strains detected in Yokohama, HMPV A2b_180nt-dup_ strains detected in Spain, and HMPV A2b_111nt-dup_ strains detected in Yokohama are indicated with filled circles in pink, blue, yellow, and green, respectively. The cluster containing HMPV A2b_180nt-dup_ and A2b_111nt-dup_ strains is indicated by a bracket.

**Fig 2:**
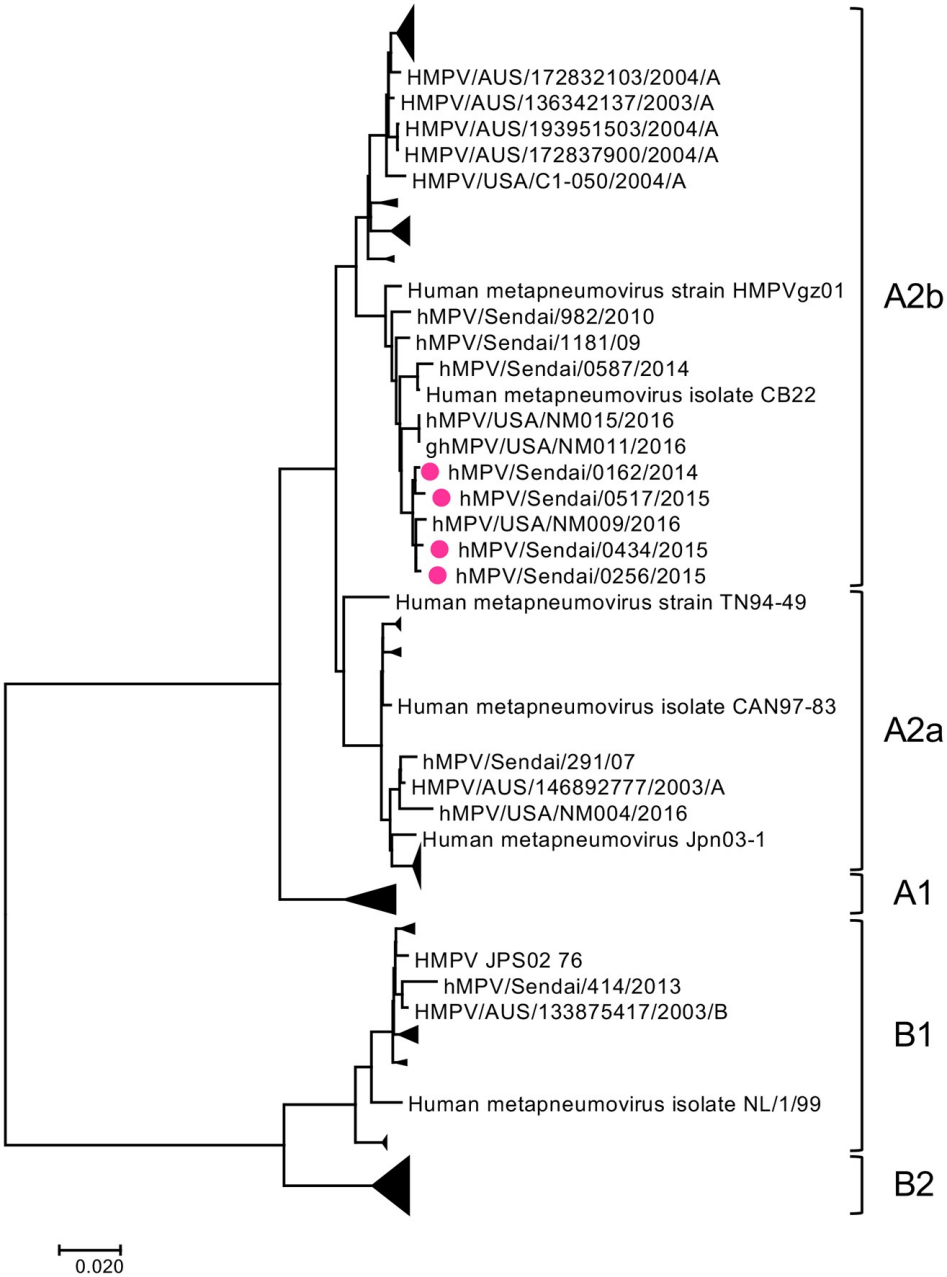
Phylogenetic tree of HMPV full genome sequence. A phylogenetic tree was constructed using the full genome sequences of 9 HMPV strains isolated in Sendai and 140 strains obtained from the NCBI nucleotide sequence database. The tree was tested with bootstrapping (100 replicates). HMPV A2b_180nt-dup_ strains detected in Sendai are indicated with filled circles in pink.

Recently, epidemiological survey studies by PCR have detected the unique 180nt-dup and 111nt-dup in the G gene of A2b subtype HMPV strains [8–10]. At the Virus Research Center, Sendai Medical Center, Japan, varieties of respiratory viruses have been routinely isolated using a microplate system harboring several types of cell lines [16, 44–46]. A2b subtype HMPV strains were isolated from 86 patients between 2014 and 2016 [16]. Among the 86 patients, 41 patients in different seasons or locations were selected, and the nucleotide sequences spanning the G gene of HMPV strains in these patients were directly determined using the clinical samples. From these clinical samples, 20 A2b_180nt-dup_ strains were detected, while no A2b strains with 111nt-dup (A2b_111nt-dup_) were detected. The information of HMPV strains isolated from the 41 patients was also provided in S2 Table. To understand the relationships of these 20 A2b_180nt-dup_ strains among HMPV strains, a phylogenetic analysis was performed using the G gene sequences of the 41 HMPV strains and previously reported HMPV strains. In the phylogenetic tree, the 20 A2b_180nt-dup_ strains detected in Sendai were located in a cluster together with 3 A2b_111nt-dup_ and 24 A2b_180nt-dup_ strains previously detected in Yokohama city, Japan, and Barcelona city, Spain [8–10] (Fig 1). These data showed that all the A2b_180nt-dup_ and A2b_111nt-dup_ strains detected in different areas are closely related to one another. Six HMPV A2b strains without nt-dup duplication in the G gene (classical A2b [A2b_classical_] strains) were also observed in this cluster (Fig 1). To obtain the highest resolution of phylogenetic relationships of subtype A2b strains, a phylogenetic analysis of the full-length genome sequences was performed. Subtype A2b HMPV strains, which were subjected to full-length genome sequencing in this study, are listed in Table 1 (Strain #1–7). The full-length genome sequence of hMPV/Sendai/0256/2015 was successfully determined directly from the clinical specimen. For other strains, it was difficult to determine the full-length genome sequences directly from the clinical specimens. Therefore, to obtain sufficient amounts of viruses for sequencing, these isolates were passaged in selected cell lines and subjected to next-generation sequencing to determine full-length genome sequences. Cell lines used for isolation and propagation of each HMPV strain are indicated in Table 1. In addition to these HMPV strains, all the full genome sequences of HMPV strains available at the NCBI (as of 20 August 2018), except for the sequences with ambiguous nucleotide(s), were used in this analysis. The A2b_180nt-dup_ strains isolated in Sendai formed a small cluster together with a HMPV A2b strain isolated in 2016 in Albuquerque, New Mexico, USA (HMPV/USA/NM009/2016, GenBank accession number KY474539) (Fig 2). These data indicate that the A2b_180nt-dup_ strains isolated in Sendai between 2014 and 2016 were closely related to the HMPV strain isolated in Albuquerque. However, based on the nucleotide sequence data in GenBank, the Albuquerque strain (HMPV/USA/NM009/2016) does not have 180nt-dup.

**Table 1:** HMPV strains used for sequence analyses.

Strain #	Strain name	Isolation year	Subtype	Isolation cell	Propagation cell *	Remarks
1	hMPV/Sendai/0256/2015	2015	A2b	M	M (5), VET (2)	180 nt-dup
2	hMPV/Sendai/0587/2014	2014	A2b	M	M (5)	-
3	hMPV/Sendai/0434/2015	2015	A2b	M	M (5) VET (1)	180 nt-dup
4	hMPV/Sendai/0517/2015	2015	A2b	M	M (3)	180 nt-dup
5	hMPV/Sendai/0162/2014	2014	A2b	M	M (2), VET (1)	180 nt-dup
6	hMPV/Sendai/1181/2009	2009	A2b	L	L (1), VE (2), VT (1)	-
7	hMPV/Sendai/0982/2010	2010	A2b	L	L (1), VE (2), VT (1)	-
8	IA3-2002	2002	A1	L	L, VET (1)	-
9	IA10-2003	2003	A1	L	L, VET (1)	-
10	hMPV/Sendai/0291/2007	2007	A2a	V	V (1), VE (2), VT (1)	-
11	hMPV/Sendai/0414/2013	2013	B1	L	L (1), VE (2), VT (1)	-
12	hMPV/Sendai/1414/2009	2009	B2	V	V (2), VE (2), VT (1)	-
13	hMPV/Sendai/1052/2011	2011	B2	L	L (3), VET (1)	-

* Numbers in the brackets indicate passage numbers.

M: MNT1; L: LLC-MK2; V: Vero; VE: VeroE6; VT: Vero/TMPRSS2; VET: VeroE6/TMPRSS2

### Nucleotide sequence changes of HMPV strains during isolation and passages in cell lines

**Fig 3:**
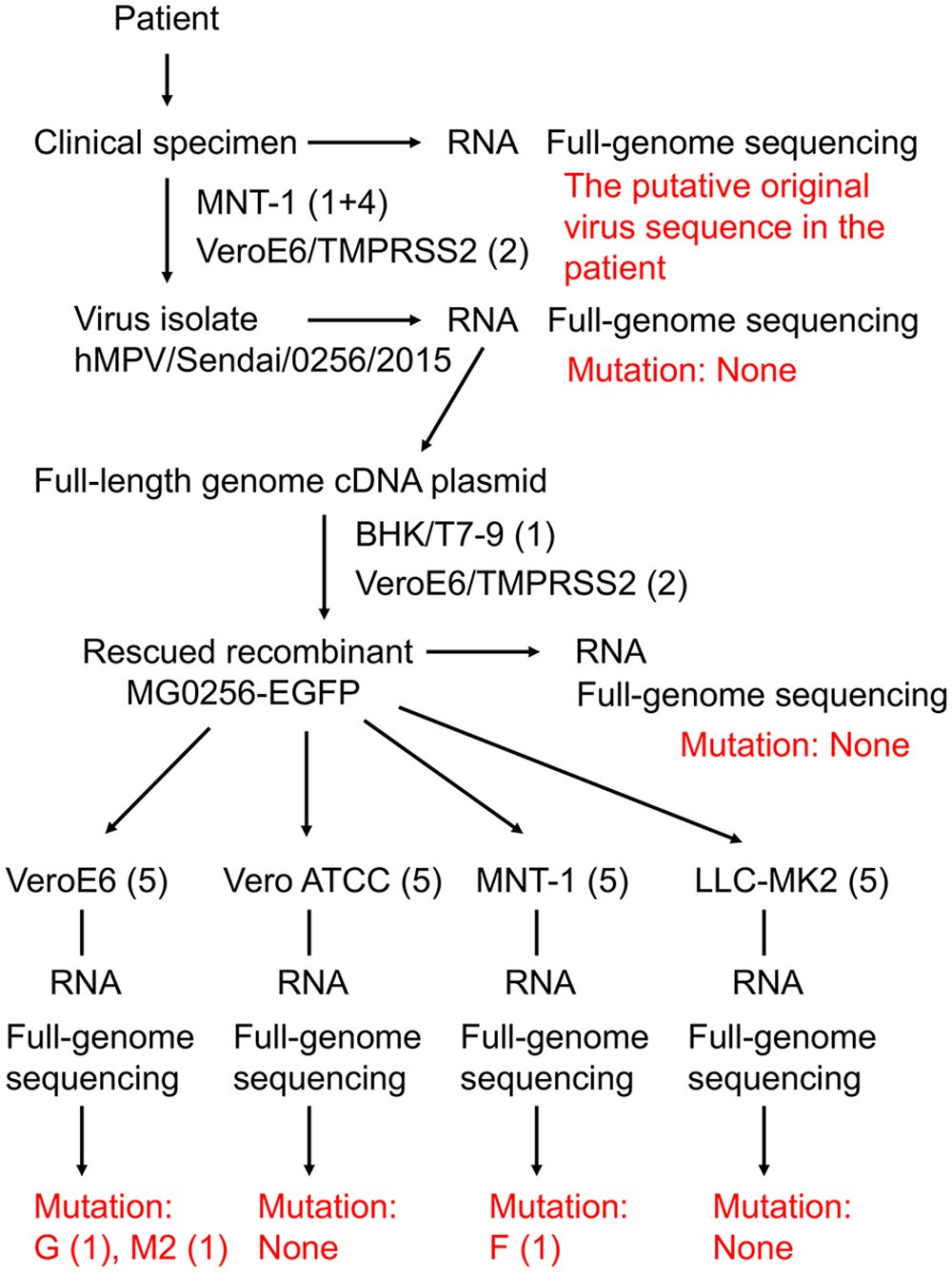
Passage history and full-genome sequence data of hMPV/Sendai/0256/2015 and its recombinant MG0256-EGFP. The hMPV/Sendai/0256/2015 was isolated using MNT-1 and VeroE6/TMPRSS2 cells. The recombinant MG0256-EGFP strain was generated based on the sequence of the hMPV/Sendai/0256/2015 strain. The full-genome virus sequence in the clinical specimen (the putative original virus sequence in the patient) and those of the isolate (hMPV/Sendai/0256/2015) and recombinant (MG0256-EGFP) after passages in various cell lines were determined. The numbers of passages in each cell line and mutations in each gene were indicated in parentheses.

Because of the limitations of small sample volume and low concentrations of viral RNAs in the samples, the full-length genome sequence was difficult to determine. However, as described above, the full-length genome sequence of hMPV/Sendai/0256/2015 was successfully determined directly from the clinical specimen (Patient #1, Table 2). The sequence would reflect the original virus sequence in the patient (Fig 3). From the clinical specimen, hMPV/Sendai/0256/2015 was isolated using MNT-1 cells, and then passaged four times in MNT-1 cells and twice in VeroE6/TMPRSS2 cells (Fig 3, Table 1). After these passages, the full-length genome sequence of hMPV/Sendai/0256/2015 was determined again and compared with the putative original virus sequence in the patient. The data showed no nucleotide change in the virus genome during the isolation and passages in MNT-1 and VeroE6/TMPRSS2 cells (Fig 3, Table 2). Based on the hMPV/Sendai/0256/2015 strain sequence, we have generated the recombinant HMPV strain, MG0256-EGFP (Fig 3). To evaluate further the effect on the induction of mutations during the propagation in cell lines, MG0256-EGFP was passaged in VeroE6, Vero ATCC, MNT-1, and LLC-MK2 cells, and the full-genome sequences of passaged strains and the parental MG0256-EGFP strain were compared. No mutation was found after the passages in Vero-ATCC and LLC-MK2 cells (Fig 3). Each one of nonsynonymous and synonymous mutations was found in the G and M2 genes, respectively, after the passages in VeroE6 cells (Fig 3, S3 Table). One nonsynonymous mutation was found in the F gene after the passages in MNT-1 cells (Fig 3, S3 Table). These data demonstrated that virus selection does not necessarily occur during the isolation and passages in MNT-1, VeroE6/TMPRSS2, Vero-ATCC, and LLC-MK2 cells, although certain selection may occur occasionally. For other four patients (Patient #2–5, Table 2), we focused on the F-M2-SH-G region, which encodes all three surface glycoproteins (F, SH, and G). The nucleotide sequence of this region was successfully determined directly from the clinical specimens of the 4 patients. From the same specimens, infectious viruses (#2–5 in Table 1) were isolated and subjected to full-length genome sequencing, as described above. These sequence data demonstrated that 2 out of the 4 strains (#3 and #4 in Tables 1 and 2) acquired no amino acid changes in all 3 surface glycoproteins (F, SH, and G) and the M2 protein during the isolation and passages in MNT-1 and VeroE6/TMPRSS2 cells (Table 1). Meanwhile, the other 2 strains (#2 and #5 in Tables 1 and 2) acquired many mutations. One strain (#2 in Tables 1 and 2) possessed highly biased U-to-C mutations in the SH and G genes. The other strain (#5 in Tables 1 and 2) had at least one amino acid change in all 3 glycoproteins and M2 protein. These data suggest that, for these two strains, certain selection or adaptation has occurred during the isolation and passages in these cell lines.

**Table 2:** Nucleotide and amino acid changes of HMPV strains during isolation and passages in cell lines.

Patient #	Virus Strain	Nucleotide and amino acid substitutions in each viral gene and protein*			
		F	M2	SH	G
1	hMPV/Sendai/0256/2015	none	none	none	none
2	hMPV/Sendai/0587/2014	none	none	TT92,93CC (L31P)	T32C (I11T)
				T102C (-)	T54C (-)
				T269C (I90T)	T82C (F28L)
				T309C (-)	TT97,98CC (L33P)
				T314C (-)	T113C (I38T)
				T341C (I114T)	T117C (-)
				T365C (I122T)	T134C (L45P)
				TT379,380CC (F127P)	T142C (Y48H)
				T420C (-)	T146C (L49P)
				T428C (L143P)	T157C (Y53H)
				T465C (-)	T198C (-)
				T482C (V161A)	T324C (-)
				T526C (Y176H)	T329C (V110A)
				T533C (I178T)	C579T (-)
				T537C (-)	
				T549C (-)	
3	hMPV/Sendai/0434/2015	none	C39T (−)	none	none
4	hMPV/Sendai/0517/2015	T953C (−)	none	none	none
5	hMPV/Sendai/0162/2014	A16G (−)	T683C(F228S)	TT92,93CC (L31P)	C470T (A157V)
		A278T (E93V)	T702C (−)	T95C (I32T)	
				T137C (V46A)	
				T472C (Y158H)	

* Nucleotide and amino acid substitutions that occurred during virus isolation and propagation are shown. Amino acid substitutions are shown in brackets, and the hyphen indicates no amino acid substitution. The first nucleotide of the initiation codon of each gene and the first methionine of each protein are deemed nucleotide position 1 and amino acid position 1, respectively.

### Infectivity of thirteen clinical HMPV isolates in different cell lines

**Fig 4:**
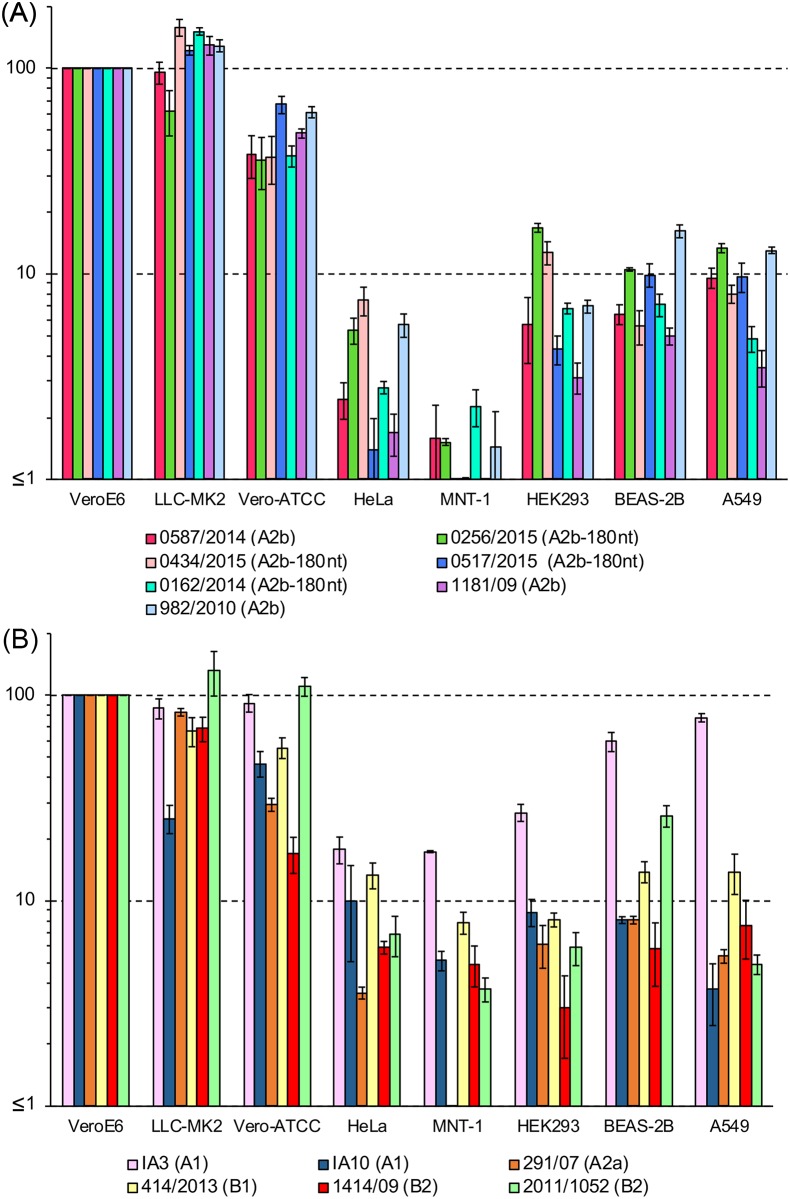
Infectivity of various cell lines with clinical isolates of HMPV. The infectivity of various cell lines (VeroE6, LLC-MK2, Vero, HeLa, MNT-1, HEK293, BEAS-2B, and A549) with thirteen clinical HMPV strains was analyzed. The numbers of HMPV infected cells in VeroE6 cells were set to 100%, and relative infectivity is presented as the average and standard errors from triplicated experiments. (A) Data of subtype A2b strains. (B) Data of subtype A1, A2a, B1, and B2 strains.

Titration of virus stocks or samples is a fundamental procedure in virus research. However, the infectivity titers may differ greatly when different cell lines are used because of the different susceptibility and permissibility of each cell line. Therefore, the most susceptible and permissible cell line is generally used for titration. In this study, to assess the cell line specificity of HMPV, the infectivity titers of HMPV were determined using eight cell lines (VeroE6, LLC-MK2, Vero-ATCC, HeLa, MNT-1, HEK293, BEAS-2B, and A549). Table 1 lists the 13 clinical isolates used for this assay. The 5 HMPV strains (#1–5 in Tables 1 and 2) that were used for sequence analyses were included in this assay. In addition to the 5 strains, 2 additional A2b_classical_ strains (#6 and #7 in Table 1) were used. In addition to these 7 A2b subtype strains, the A1, A2a, B1, and B2 subtype strains (Table 1) were included in this assay. The infectivity titers of all clinical strains were the greatest when determined using VeroE6 or LLC-MK cells (Fig 4). The infectivity titer of each strain in VeroE6 cells was set to 100% for comparison. Vero cells purchased from ATCC (Vero-ATCC cells) showed 29.4%–110.6% infectivity to these clinical isolates. Low to moderate levels of infectivity titers were detected in BEAS-2B (5.6%–60.0%), A549 (3.5%–77.9%) and HEK293 cells (3.1%–26.9%). Uniquely, IA3 strain showed significantly higher virus titers than other clinical isolates (Fig 4). As described, the titers varied among HMPV strains, but all were lower than those in VeroE6 and LLC-MK2 cells. The infectivity titers in HeLa cells were very low and varied significantly among strains (1.4%–17.8%). Unexpectedly, the infectivity titers of all the clinical isolates were very low (0.5%–7.8%) when MNT-1 cells were used for titration (Fig 4), although five of them (#1–5 in Table 1) were isolated efficiently using MNT-1 cells. It was noted that, in spite of the lower infectivity in MNT-1 cells than in other susceptible cell lines, CPE in MNT-1 was easier to detect than in VeroE6 and LLC-MK2 cells, when these cells were incubated with clinical specimens of HMPV [16]. We also speculate that there are particular differences between HMPV virions in patients and those in the supernatants of cultured cells.

### Infectivity of six recombinant HMPV^GFP^ strains in different cell lines

**Fig 5:**
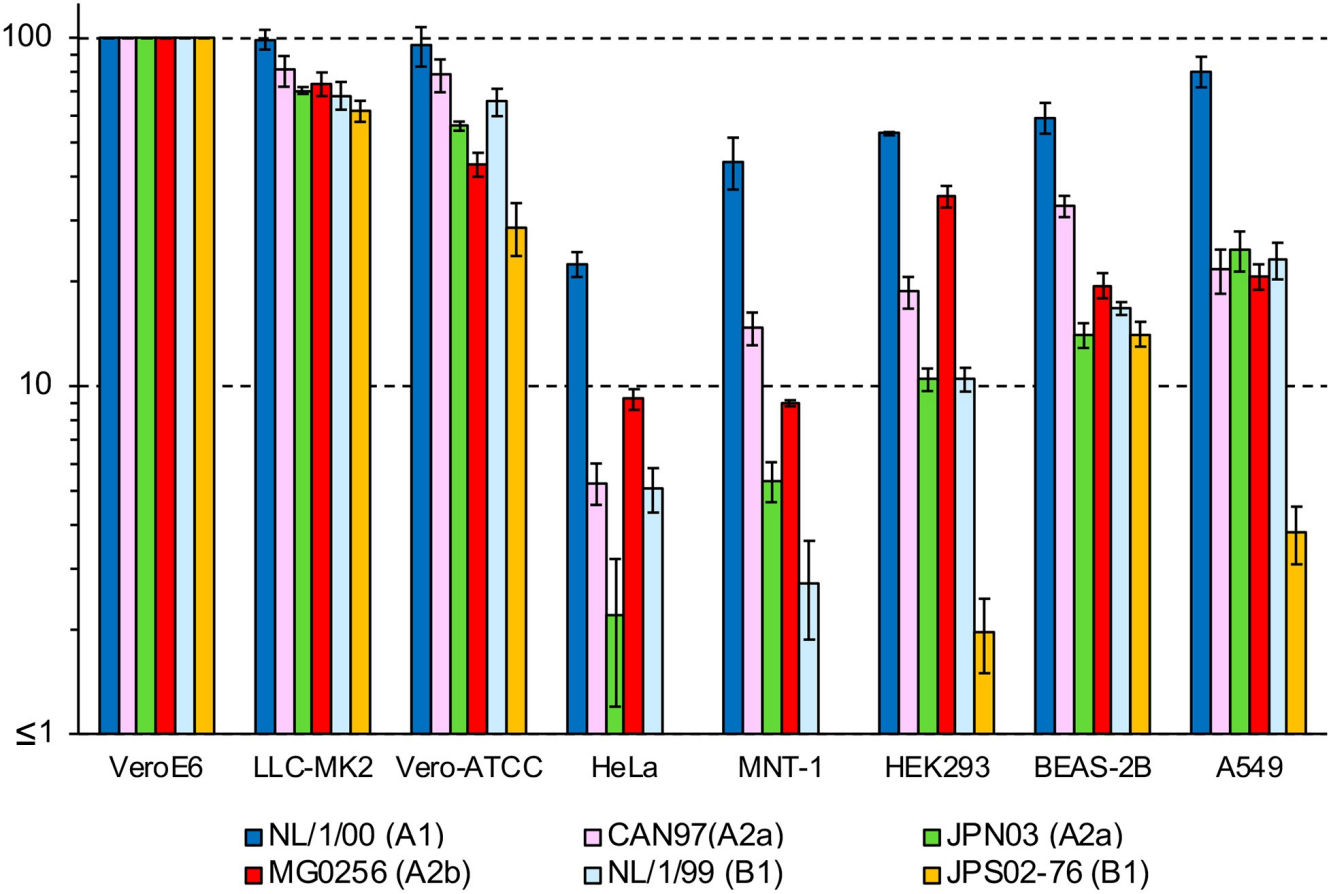
Infectivity of various cell lines with recombinant HMPV strains. The infectivity of various cell lines (VeroE6, LLC-MK2, Vero, HeLa, MNT-1, HEK293, BEAS-2B, and A549) with six recombinant HMPV^GFP^ strains was analyzed. The numbers of HMPV infected cells in VeroE6 cells were set to 100%, and relative infectivity is presented as the average and standard errors from triplicated experiments.

**Fig 6:**
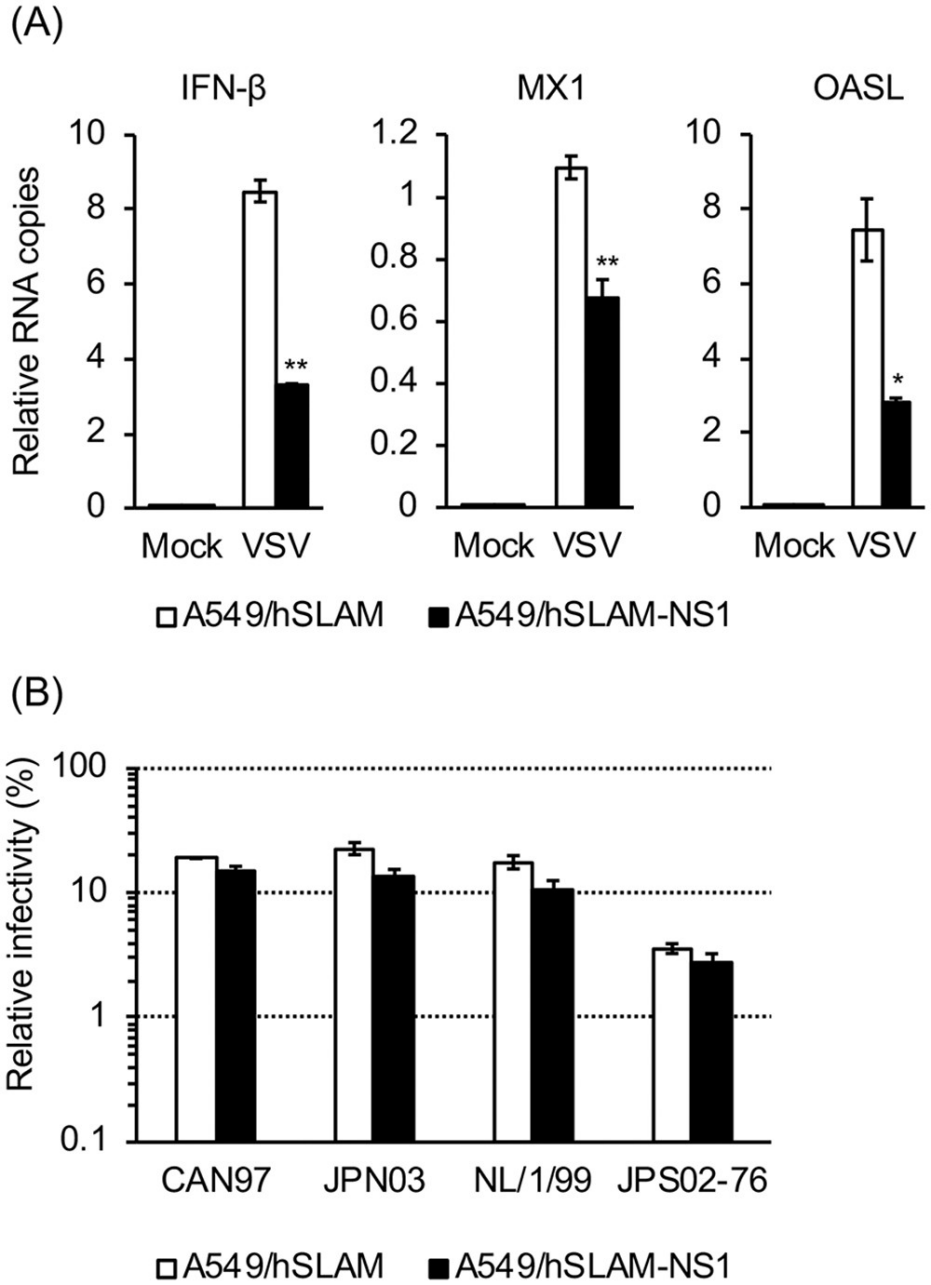
Infectivity of A549/hSLAM and A549/hSLAM-NS1 cells with HMPV strains. (A) A549/hSLAM and A549/hSLAM-NS1 cells were infected with VSV at an MOI of 1.0. At 6 hpi, total RNAs were extracted from VSV-infected or mock-infected cells. The levels of mRNA of IFN-β, MX1, and OASL were determined by qPCR, and the values were normalized to levels of the control gene HPRT1. Relative RNA copies are presented as the average and standard errors from triplicated experiments. Statistical significance was calculated using Student’s t test (*P < 0.05; **P < 0.01). (B) The infectivity of VeroE6 and 2 types of A549 (A549/hSLAM and A549/hSLAM-NS1) cells with 4 recombinant HMPV^GFP^ strains was analyzed. The numbers of HMPV-infected cells in VeroE6 cells were set to 100%, and relative infectivity is presented as the average and standard errors from triplicated experiments.

Many studies have been conducted using recombinant HMPV strains. Therefore, a comparison between the clinical isolates and the recombinant HMPV strains was important. Table 3 shows a list of six recombinant HMPV^GFP^. The parental strains of JPS02-76EGFP (JPS02-76^GFP^), NL/1/99-gfp (NL/1/99^GFP^), and NL/1/00-gfp (NL/1/00^GFP^) have been isolated and propagated in tMK cells [4, 11, 21, 23, 47], while those of rHMPV-Rluc/GFP (JPN03-1^Rluc/GFP^) and MPV-GFP1 (CAN97-83^GFP^) have been isolated and propagated in LLC-MK2 cells [20, 22, 26, 48]. The parental strain of MG0256-EGFP (MG0256^EGFP^) was isolated and propagated in MNT-1 and VeroE6/TMPRSS2 cells (Tables 1 and 3). JPS02-76^GFP^, NL/1/99^GFP^, NL/1/00^GFP^, JPN03-1^Rluc/GFP^, and MG0256^EGFP^ were generated from the full-length genome plasmids using BHK/T7-9 cells, as described previously [21], and propagated in VeroE6/TMPRSS2 cells. CAN97-83^GFP^ was purchased from ViraTree (http://www.viratree.com/) and propagated in VeroE6/TMPRSS2 cells. The subtypes of the parental strains of six recombinant strains are shown in Table 3. The infectivity titers of all six HMPV^GFP^ strains were greatest when they were determined using VeroE6 cells (Fig 5). The infectivity titer of each HMPV^GFP^ strain in VeroE6 cells was set to 100% for comparison (Fig 5). All the six HMPV^GFP^ strains showed reasonably high titers in LLC-MK2 cells (61.8%–99.3%). The titers in Vero-ATCC cells were also relatively high, as observed with the clinical isolates of HMPV. Among the six recombinant HMPV^GFP^ strains, the cell line specificity was the strictest for JPS02-76^GFP^ [21]. The infectivity titer of JPS02-76^GFP^ determined using HeLa, MNT-1, HEK293, and A549 cells was only 0.7%, 0.4%, 2.0%, and 3.8%, respectively. In contrast, all tested cells showed reasonably high infectivity with NL/1/00^GFP^ strain. The infectivity titer of NL/1/00^GFP^ using HeLa, MNT-1, HEK293, and A549 cells was 22.4%, 44.3%, 53.2%, 59.2%, and 80.4%, respectively. The infectivity titer of other four HMPV^GFP^ strains were intermediate between those of JPS02-76^GFP^ and NL/1/00^GFP^, ranging 2.2%–9.2% in HeLa cells, 2.7%–14.7% in MNT-1 cells, 10.5%–35.1% in HEK293 cells, 14.0%–32.9% in BEAS-2B cells, and 20.7%–24.6% in A549 cells (Fig 5). A lack of the type I interferon (IFN) gene [49] is a possible reason for why HMPV showed the highest infectivity titers in VeroE6 cells. We have tested whether blocking the innate immune signals enhances the infectivity of HMPV. For this purpose, A549 cells constitutively expressing influenza A virus NS1 protein (A549/hSLAM-NS1) were used. NS1 protein is known to inhibit IFN induction and/or signaling [50, 51]. As expected, VSV-infected A549/hSLAM-NS1 cells showed significantly lower transcription levels of IFN-β, MX1, and OASL, compared with VSV-infected parental (NS1-nontransduced) A549/hSLAM cells (Fig 6A). However, the infectivity titers of four HMPV^GFP^ strains determined using A549/hSLAM-NS1 cells were mostly unchanged, when compared with those using A549/hSLAM (Fig 6B), suggesting that the lack of IFN responses is not the sole determinant of the high infectivity of VeroE6 cells with HMPV.

**Table 3:** List of GFP-expressing recombinant HMPV strains.

Parental strain						Recombinant strain			
Name	Isolation cell	Propagation cell	Isolation year	Subtype	Reference	Name	rescue cell	Propagation cell	Reference
NL/1/00	tMK	tMK	2001	A1	[4]	NL/1/00-gfp	BHK/T7-9	VET	[23]
CAN97-83	L	L, V	2003	A2a	[26]	MPV-GFP1	BSR T7/5	L, VET	[20]
JPN03-1	L	L	2003	A2a	[48]	rHMPV-Rluc/GFP	BHK/T7-9	VET	[22]
hMPV/Sendai/0256/2015	M	M, VET	2015	A2b	This study	MG0256-EGFP	BHK/T7-9	VET	This study
NL/1/99	tMK	tMK	2003	B1	[4]	NL/1/99-gfp	BHK/T7-9	VET	[23]
JPS02-76	tMK	tMK, V	2002	B1	[11]	JPS02-76EGFP	BHK/T7-9	VET	[21]

L: LLC-MK2; V: Vero; VET: VeroE6/TMPRSS2; tMK: tertiary monkey kidney

## Discussion

The analysis of cell line specificity for clinical and recombinant HMPV strains demonstrated that VeroE6 cells generally showed the highest infectivity with HMPV strains. The infectivity of Vero-ATCC was also high, but it was 2- to 3-fold lower than that of VeroE6 cells. Vero and Vero-derived cell lines have a large deletion in the gene cluster of the interferon (IFN) genes [52]. Thus, these cells do not produce type I IFNs and show high susceptibility to many kinds of viruses [53, 54]. Lack of IFN responses could be a major factor for VeroE6 cells having high infectivity with HMPV. However, suppression of the innate immune responses, including IFN production, by the influenza virus NS1 protein did not enhance the infectivity of A549 cells with HMPV at all. LLC-MK2 cells also demonstrated similarly high infectivity with clinical HMPV strains as did VeroE6 cells. Unlike VeroE6 cells, LLC-MK2 cells retain a functional IFN system and produce IFN in response to virus infections and exhibit an antiviral state [55–58]. Therefore, the quality or condition of the IFN system is not the main factor that determines the infectivity of each cell line with HMPV.

Considering the data of numerous studies [1, 3, 18, 19, 24–28], the consensus is that there is high infectivity in VeroE6 and LLC-MK2 cells for all HMPV strains. When compared with VeroE6 and LLC-MK2 cells, BEAS-2B, A549, HEK293, MNT-1, and HeLa cells showed significantly lower infectivity with HMPV. In addition, the infectivity of these cell lines varied significantly among HMPV strains. Among the thirteen clinical isolates, only IA3 (subtype A1) strain showed more than 10% infectivity in all five cell lines (BEAS-2B, A549, HEK293, MNT-1, and HeLa cells). In addition, among the six recombinant HMVP^GFP^ strains, NL/1/00^EGFP^ (subtype A1) showed the highest infectivity in these five cell lines (BEAS-2B, A549, HEK293, MNT-1, and HeLa cells). However, the high infectivity phenotype in these cell lines was not a common feature for subtype A1 strains, because another subtype A1 strain (IA10) showed as low infectivity as other clinical isolates in these five cell lines. These variations in cell line specificity may reflect the different behaviors among the HMPV strains in the virus life cycle, such as receptor specificity [59, 60], entry route [31], and fusion triggering mechanism [61]. However, it should be noted that no significant difference has been observed in clinical presentations among HMPV strains [62].

The variations among HMPV strains may be produced by mutations during isolation and passages of HMPV strains in specific cell lines. However, this study demonstrated that no amino acid change was required in the surface glycoproteins or even in the entire genome of HMPV to propagate in VeroE6/TMPRSS2 and MNT-1 cells. The F protein is responsible for viral attachment and membrane fusion and is essential for viral infectivity. Compared with the F protein, the roles of the SH and G proteins are less clear, because these proteins are dispensable for virus infectivity [63]. The SH protein has properties consistent with those of viroporins and modulates viral fusogenic activity [64]. The G protein of certain HMPV strains binds to cell surface glycosaminoglycan and may enhance viral attachment to host cells [65, 66]. HMPV G protein binds to the RIG-I cytoplasmic RNA recognition receptor and inhibits the expression of type I IFN and proinflammatory cytokines of infected cells [67]. Even though no adaptation mutation was necessary for the viral glycoproteins to grow in the cell lines, certain strains have acquired mutations. No common mutation was observed, but the unique observation was that the nucleotide changes were mostly U to C, mainly in SH and G genes. Similar nucleotide changes have been reported for several viruses as a result of RNA editing by adenosine deaminases [68]. It has also been documented that a high rate mutation due to adenosine deaminases occurs on defective interfering RNA of HMPV, when the virus is passaged at high MOI [69].

Recently, unique HMPV A2b_180nt-dup_ and A2b_111nt-dup_ strains have been detected [8–10]. Previous studies [8–10] have only detected the virus genomes in clinical samples by RT-PCR, while this study showed isolated infectious HMPV A2b_180nt-dup_ strains. No A2b_111nt-dup_ strains were detected in this study. The full-genome sequence analysis of A2b_180nt-dup_ strains demonstrated that 180nt-dup in the G gene was maintained in the virus genome during the process of virus isolation and passages in cell lines. No nt-dup was detected in other genes. The different length of the G gene was also reported in avian metapneumovirus (AMPV) subtype C, the most closely related virus to HMPV among all the known viruses [70–72]. Extensive passages of AMPV subtype C with the long G gene in Vero cells have resulted in almost complete deletion of the G gene from the viral genome [73]. Nucleotide duplications in the G gene have also been reported in human respiratory syncytial virus (RSV) [74, 75]. The nucleotide duplication (60 nucleotides in length) in the G gene of RSV enhances the virus attachment to CHO-K1 and BEAS-2B cells [74, 76]. The G protein of certain HMPV strains supports virus attachment to host cells by binding to cell surface glycosaminoglycan [65, 66]. However, the cell line specificity of HMPV A2b_180nt-dup_ strains remained similar to that of the HMPV A2b_classical_ strains.

In this study, we re-evaluated and compared the cell line specificity of clinical isolates and recombinant HMPV strains, because accumulated data have resulted in complications for understanding the cell line specificity of HMPV. Our data in this study demonstrate that VeroE6 and LLC-MK2 cells show the highest infectivity with clinical isolates and commonly used recombinant HMPV strains, including the novel A2b_180nt-dup_ strains, five recombinant HMPV^GFP^, which have been used in different laboratories, and the newly generated recombinant HMPV A2b_180nt-dup_ strain, MG0256-EGFP. The consensus is that VeroE6 and LLC-MK2 cells have the highest infectivity for all the HMPV strains. Meanwhile, the infectivity of other cell lines (BEAS-2B, A549, HEK293, MNT-1, and HeLa cells) with HMPV is significantly lower than that of VeroE6 and LLC-MK2 cells and varies significantly among HMPV strains. These variations are observed for both clinical isolates and recombinant HMPV strains and not related to HMPV subtypes, cell lines used for isolation and propagation, specific genome mutations, or nucleotide insertions. Therefore, these variations would be intrinsic to HMPV strains, but the significance in HMPV infection in humans is unclear because no significant difference has been observed in clinical presentations among HMPV strains [62].

## Supporting information

S1 Dataset Gene sequences of HMPV strains analyzed in this study. (FAS)

S2 Dataset Full genome sequences of HMPV strains analyzed in this study. (FAS)

S1 Table HMPV strains used in this study. (DOCX)

S2 Table Primers and Universal ProbeLibrary probes used for quantitative PCR. (DOCX)

S3 Table Nucleotide and amino acid changes of MG025-EGFP strain during passages in each cell line. (DOCX)
