# Supplementary material for: Consensus and variations in cell line specificity among human metapneumovirus strains
Source: PLoS One. 2019 Apr 23;14(4):e0215822. doi: 10.1371/journal.pone.0215822 (PMC6478314; doi:10.1371/journal.pone.0215822)
Supplement: S1 Table — (DOCX) [file pone.0215822.s003.docx]

S1 Table

| Target gene | Primer sequence (5' to 3') | UPL probe |
| --- | --- | --- |
| HPRT1 | GGGAGGCCATCACATTGTAG | Probe 62 |
|  | CACTATTTCTATTCAGTGCTTTGA |  |
| IFN-β | CTTTGCTATTTTCAGACAAGATTCA | Probe 20 |
|  | GCCAGGAGGTTCTCAACAAT |  |
| MX1 | ACCTGATGGCCTATCACCAG | Probe 5 |
|  | TGAAGAACTGGATGATCAAAGG |  |
| OASL | ATGTTGGACGAAGGCTTCAC | Probe 68 |
|  | TTGGTCCAGTAGATACAGATGACTTC |  |
