# Supplementary material for: Consensus and variations in cell line specificity among human metapneumovirus strains
Source: PLoS One. 2019 Apr 23;14(4):e0215822. doi: 10.1371/journal.pone.0215822 (PMC6478314; doi:10.1371/journal.pone.0215822)
Supplement: S2 Table — (DOCX) [file pone.0215822.s004.docx]

S2 Table

| Strain name | Isolation cell | Remarks |
| --- | --- | --- |
| hMPV/Sendai/0162/2014 | MNT1 | 180nt-dup |
| hMPV/Sendai /0168/2014 | LLC-MK2 |  |
| hMPV/Sendai /0170/2014 | LLC-MK2 | 180nt-dup |
| hMPV/Sendai /0180/2014 | LLC-MK2 |  |
| hMPV/Sendai /0226/2014 | MNT1 |  |
| hMPV/Sendai /0238/2014 | MNT1 |  |
| hMPV/Sendai /0242/2014 | LLC-MK2 |  |
| hMPV/Sendai /0243/2014 | MNT1 |  |
| hMPV/Sendai /0254/2014 | LLC-MK2 | 180nt-dup |
| hMPV/Sendai /0584/2014 | LLC-MK2 |  |
| hMPV/Sendai /0587/2014 | MNT1 |  |
| hMPV/Sendai /0625/2014 | MNT1 |  |
| hMPV/Sendai /0629/2014 | MNT1 |  |
| hMPV/Sendai /0699/2014 | MNT1 |  |
| hMPV/Sendai /0700/2014 | MNT1 |  |
| hMPV/Sendai /0825/2014 | LLC-MK2 |  |
| hMPV/Sendai /0827/2014 | LLC-MK2 |  |
| hMPV/Sendai /0106/2015 | MNT1 |  |
| hMPV/Sendai /0107/2015 | MNT1 |  |
| hMPV/Sendai /0112/2015 | LLC-MK2 |  |
| hMPV/Sendai /0219/2015 | MNT1 |  |
| hMPV/Sendai /0220/2015 | MNT1 |  |
| hMPV/Sendai /0223/2015 | MNT1 | 180nt-dup |
| hMPV/Sendai /0239/2015 | MNT1 | 180nt-dup |
| hMPV/Sendai /0251/2015 | MNT1 | 180nt-dup |
| hMPV/Sendai /0256/2015 | MNT1 | 180nt-dup |
| hMPV/Sendai /0258/2015 | MNT1 | 180nt-dup |
| hMPV/Sendai /0261/2015 | MNT1 |  |
| hMPV/Sendai /0274/2015 | MNT1 | 180nt-dup |
| hMPV/Sendai /0331/2015 | LLC-MK2 | 180nt-dup |
| hMPV/Sendai /0338/2015 | LLC-MK2 | 180nt-dup |
| hMPV/Sendai /0434/2015 | MNT1 | 180nt-dup |
| hMPV/Sendai /0447/2015 | MNT1 | 180nt-dup |
| hMPV/Sendai /0517/2015 | MNT1 | 180nt-dup |
| hMPV/Sendai /0539/2015 | MNT1 |  |
| hMPV/Sendai /0600/2015 | MNT1 | 180nt-dup |
| hMPV/Sendai /1305/2015 | MNT1 | 180nt-dup |
| hMPV/Sendai /0115/2016 | MNT1 | 180nt-dup |
| hMPV/Sendai /0174/2016 | MNT1 | 180nt-dup |
| hMPV/Sendai /0468/2016 | LLC-MK2 | 180nt-dup |
| hMPV/Sendai /0633/2016 | MNT1 | 180nt-dup |
