# Supplementary material for: Consensus and variations in cell line specificity among human metapneumovirus strains
Source: PLoS One. 2019 Apr 23;14(4):e0215822. doi: 10.1371/journal.pone.0215822 (PMC6478314; doi:10.1371/journal.pone.0215822)
Supplement: S3 Table — (DOCX) [file pone.0215822.s005.docx]

S3_Table Nucleotide and amino acid changes of MG0256-EGFP strains during passages in VeroE6, Vero ATCC, MNT-1, and LLC-MK2 cells

| Passaged cell* | Nucleotide and amino acid changes of MG025-EGFP strain during passages in each cell line ** | | | | | | | |
| --- | --- | --- | --- | --- | --- | --- | --- | --- |
|  | N | p | M | F | M2 | SH | G | L |
| VeroE6 | none | none | none | none | T669C (-) | none | G62C (R21P) | none |
| Vero ATCC | none | none | none | none | none | none | none | none |
| MNT-1 | none | none | none | C1446A (S482R) | none | none | none | none |
| LLC-MK2 | none | none | none | none | none | none | none | none |

* Parental MG0256-EGFP strain (Table 3) was passaged 5 times in VeroE6, Vero ATCC, MNT-1, or LLC-MK2 cells with a low MOI (MOI<0.1).

** Amino acid substitutions are shown in brackets, and the hyphen indicates no amino acid substitution. The first nucleotide of the initiation codon of each gene and the first methionine of each protein are deemed nucleotide position 1 and amino acid position 1, respectively.
